# Retrospective study on the use of lidocaine constant rate infusions for the treatment of ileus in ruminants and camelids

**DOI:** 10.1111/jvim.16262

**Published:** 2021-09-13

**Authors:** Katie Yau, Jennifer Halleran, Melanie Boileau, Derek Foster

**Affiliations:** ^1^ Department of Population Health and Pathobiology, College of Veterinary Medicine NC State University Raleigh North Carolina USA; ^2^ Department of Veterinary Clinical Sciences, College of Veterinary Medicine Oklahoma State University Stillwater Oklahoma USA

**Keywords:** ileus, lidocaine, prokinetic, ruminants

## Abstract

Limited knowledge exists regarding the use of lidocaine as a prokinetic in ruminants and camelids to treat gastrointestinal ileus. In this retrospective study, ruminant and camelid cases diagnosed with ileus and treated with a lidocaine constant rate of infusion were assessed for adverse reactions and medical outcomes. A review of medical records was performed to identify cases in which lidocaine was administered as a prokinetic. Ten cases were identified consisting of 8 cattle, 1 goat, and 1 alpaca. Nine animals improved with a lidocaine treatment. No adverse effects were reported during lidocaine administration. Nine animals were discharged, and 1 was euthanized.

**Abbreviations**
CRI: constant rate of infusion
POI: postoperative ileus

## 1 INTRODUCTION

Lidocaine is a commonly used medication in veterinary medicine as a local anesthetic, but also as an anti‐arrhythmic, anti‐inflammatory and prokinetic agent.^1^, ^2^, ^3^, ^4^ Typically, in equids, the prokinetic properties of lidocaine are used to treat postoperative ileus (POI).^2^, ^5^ However, despite routine use for POI, there is conflicting evidence to support its efficacy in improving motility and survival rates.^1^, ^2^, ^6^, ^7^, ^8^, ^9^, ^10^ Additionally, the mechanism of action for treating POI is not well understood and might include a combination of anti‐inflammatory or analgesic effects, and ability to stimulate or block intestinal muscular contraction.^6^ Other reports suggest the role in mitigating POI is through reducing gastric reflux and decreasing intraluminal jejunal and peritoneal fluid accumulation.^11^ There are few available case reports of ileus as a condition in ruminants, but it is mostly characterized as a postoperative occurrence after surgical handling of the intestines.^12^

Research into the use of prokinetic medications in ruminants has focused on abomasal motility and there is a need to investigate agents that affect intestinal motility. Therefore, there is a need for a more suitable alternative to treat intestinal ileus in ruminants, such as lidocaine.

Currently, there is a lack of research describing the efficacy of lidocaine as an intestinal prokinetic medication in ruminants. Specific research indicating the safety and efficacy of lidocaine as a prokinetic is needed to guide use in these species. In this retrospective case series, the medical records of ruminants and camelids with ileus treated with lidocaine were analyzed. The objective was to determine whether the lidocaine constant rate of infusion (CRI) dose and rate for use in postoperative ileus in horses had any adverse side effects when administered in ruminants and camelids, and the clinical outcome after lidocaine administration.

## 2 MATERIALS AND METHODS

The search for relevant cases began by requesting cases from Oklahoma State University. A search was conducted of appropriate cases with the Universal Veterinary Information System (UVIS, Atlanta, Georgia). Searches for bovine, ovine, caprine, and camelid cases with lidocaine charged to their bills was conducted. Further searching for ruminants and camelids with any of the following diagnoses: ileus, intussusception, and right displaced abomasum was performed as some belief that these might have received a lidocaine CRI. Each case was then analyzed for the predetermined inclusion criteria.

The inclusion criteria included either a stated or presumptive diagnosis of ileus. Clinical scenarios given a diagnosis of ileus included: a stated diagnosis of ileus; an abdominal ultrasound finding of hypomotile or nonmotile intestines; an abdominal ultrasound finding of gas‐distended or dilated small intestines with a distended abdomen, or painful abdomen, or both; and abdominal distension with lack of fecal production or feces found on rectal exam. In addition, historical information provided by the owner was reviewed and included if consistent with clinical signs of ileus (abdominal distention, decreased to absent fecal production).

Only cases administered lidocaine for the treatment of ileus were included in the study. Cases where lidocaine was administered for a prophylactic or different therapeutic purpose were excluded.

The case information collected came from complete review of the medical record. Species, breed, sex, age, duration of hospital stay, presenting complaint, diagnosis, lidocaine CRI dose and rate, subjective (abdominal distension and fecal output) and objective (ultrasound findings, duration of hospitalization) assessments of the species after the start of the CRI treatment, adverse effects after the start of the CRI treatment, and ancillary treatments were recorded for each case. Adverse effects that were monitored including muscle tremors, seizure activity, tachycardia, and arrhythmia.

## 3 RESULTS

The initial search process through Oklahoma State University yielded 11 cases. Two cases were removed because of lidocaine administration as an analgesic, and 1 because of administration as an anti‐arrhythmic. The search through North Carolina State University yielded 2 cases that met the inclusion criteria. The total number of cases assessed in this study was 10 over a 11‐year period (2009‐2020).

### 3.1 Signalment

For the 10 cases that met the inclusion criteria, there were 8 cattle, 1 alpaca, and 1 goat. Of the 8 cattle, 7 were beef cattle, and 1 was a dairy calf. The sex distribution consisted of 5 males and 5 females. The age distribution ranged from 1 day to 9 years, with a median age of 1.75 years.

### 3.2 Presenting complaints and clinical diagnoses

The presenting complaints and in‐hospital diagnoses are displayed in Supplemental Table S1. The presenting complaints were colic (5/10), abdominal distension/bloat (3/10; 1 of which also presented with colic), weakness/lethargy (2/10), and distended small intestines on rectal exam (1/10). Ileus was the most common primary diagnosis (4/10) followed by 1 case of each of the following: cecal dilatation (also included in ileus count), ceco‐colic volvulus, failure of passive immunity (also included in ileus count), focal peritonitis, small intestinal intussusception, enteritis with spiral colon impaction, septicemia, and intestinal adhesions. Ultrasonographic findings and selected clinicopathologic results are detailed in Supplemental Table S2.

### 3.3 Surgical treatment

Eight out of the 10 animals had abdominal exploratory surgeries before the lidocaine treatment. Supplemental Table S3 provides the surgery performed and findings.

### 3.4 Lidocaine constant rate of infusion therapy

Both hospitals utilized a loading dose of 1.3 mg/kg lidocaine HCl 2% followed by a CRI rate of 0.05 mg/kg/minute. The loading dose of lidocaine was administered as a bolus. Depending upon the case, lidocaine was diluted into an appropriate isotonic replacement fluid and administered at a rate to meet their fluid needs with either a fluid pump or a syringe pump. The duration of lidocaine therapy for each patient ranged from 1 to 3 days (median 2.5 days). The duration of hospitalization ranged from 2 to 7 days (median 4.5 days). In order to assess the success of the lidocaine treatment, improvement or resolution of clinical signs after the start of the treatment was assessed in the clinical notes. The clinical signs assessed to determine improvement included: changes in mentation (bright and alert), increased appetite, normal fecal production, minimal abdominal distention, and present intestinal motility observed via ultrasound. Based on the above clinical signs, 8 cases had positive outcomes (positive outcomes were cases discharged from the hospital), whereas 2 cases had no improvement in clinical signs. Of the 2 cases with no improvement, 1 was euthanized at the end of its hospital stay, and the other had worsening of clinical signs (increased intestinal distention) on the second day of lidocaine therapy, but ultimately improved and was discharged. Lidocaine was discontinued in this case because of worsening of clinical signs‐increased pain, distended firm abdomen and no fecal production. With these clinical signs, surgery was performed within an hour of presenting and an abnormal band was identified from the caudal greater omentum. Supplemental Table S4 provides ancillary treatments.

### 3.5 Adverse effects

There were no adverse events including neurologic or cardiovascular effects that were attributable to the lidocaine treatments identified in the medical records for any case. Neurologic adverse effects include agitation, restlessness, muscle fasciculation, and seizure activity. Cardiovascular effects included tachycardia and arrhythmia.

### 3.6 Case outcome

Nine of the 10 cases were ultimately discharged, and 1 was euthanized. For the euthanized species, the ileus was unresolved after 5 days of hospital care. Follow‐up calls with clients were not completed for discharged animals, so no long‐term outcomes were assessed.

## 4 DISCUSSION

There is scarce research reporting the use of lidocaine as a treatment for ileus in ruminants and camelids in either research or clinical settings. With the extensive use of lidocaine to treat POI in horses, the objective was to determine the medical outcome and any adverse effects noted. Lidocaine CRI was evaluated as a treatment option in this retrospective case series. No adverse effects were recorded, the majority had positive outcomes with 9 out of 10 discharged from the hospital. The findings of this retrospective study indicate this lidocaine CRI treatment regimen was safe to use in this group of hospitalized cases with apparent resolution of clinical signs of ileus.

The lidocaine loading dose and CRI rate was extrapolated from equine medicine for the treatment of POI.^1^, ^2^, ^13^, ^14^, ^15^ The loading dose and CRI rate had previously been determined through pharmacokinetic studies of plasma lidocaine concentrations while monitoring signs of toxicity in horses. While there is a reported toxic dose of lidocaine in goats at 5 mg/kg and cattle at 10 mg/kg and it is believed to be less than horses, the lack of a true safe dosing range requires close assessment of this treatment to ensure it is safe.^16^ Our study assessed the safety of this regimen by reviewing all aspects of the records for any adverse reactions, and we did not identify any adverse effects that could be attributed to lidocaine toxicity. The findings of this retrospective study indicate this lidocaine CRI treatment regimen was not associated with neurological or cardiovascular adverse effects in this group of hospitalized cases with apparent improvement in clinical sign. This led to the conclusion that the lidocaine loading dose and CRI rate used in horses appears to be safe when extrapolated to ruminants.

The information in the literature regarding lidocaine administration to horses for POI is conflicting. Numerous studies demonstrate benefit in alleviating ileus, whereas other studies provide inconclusive results and conclusions.^1^, ^2^, ^11^, ^17^ The measures by which these studies evaluated efficacy included the duration of gastric reflux, survival rate, and duration of hospital stay.^1^, ^11^ There are few studies or case reports available in ruminants utilizing lidocaine. One study investigating hemorrhagic bowel syndrome in dairy cattle utilized lidocaine administered IV as a therapy option; animals treated medically in this study died.^10^ There is a case report of a bull being treated for hemorrhagic bowel syndrome that did receive IV infusions of lidocaine.^18^ For this bull, the use of lidocaine was tolerated with no adverse effects observed. This bull ultimately survived with aggressive medical therapy. The authors of this case report hypothesize that the multiple benefits of lidocaine such as its prokinetic ability, antimicrobial activity and effects on neutrophil function, allowed for a positive outcome in this case. A separate case reported use of lidocaine CRI successfully in a cria with cryptosporidiosis.^19^ In this case series, only 1 animal had clear evidence of enteritis, so this antimicrobial effect could have played a role in the positive outcome in this case.^20^, ^21^ An additional study observed the effect of different prokinetic agents on POI with motilin and ghrelin as motility markers.^22^ They observed that the use of lidocaine resulted in significantly higher motilin concentrations, suggesting it might be useful for lambs with POI. Our study assessed the efficacy of this therapy by identifying evidence of improved intestinal motility in the medical record after initiation of the CRI. In the 9 cases with a positive outcome, 8 had an improvement or resolution of clinical signs after treatment with lidocaine. This suggests a positive benefit of lidocaine in these cases of ileus; however, because of the retrospective nature of this study, this could be coincidental, secondary to some other effect of lidocaine, or biased by the interpretation of the attending clinician.

Abdominal exploratory surgery was performed on most of the animals (7/10). In addition to the primary disease in these cases, the surgical procedure might have contributed to the incidence of ileus because of the anesthetic and analgesic drugs given and the manipulation of the intestines during surgery. This connection highlights the potential benefit of instituting this treatment after abdominal surgeries as a preemptive measure to avoid the development of ileus. This case series evaluated lidocaine as a treatment rather than a preventative therapy.

In the United States, lidocaine is labeled for the use of local anesthesia in food producing species. Utilizing lidocaine as a prokinetic would be considered an extra label drug use. This would then entail contacting the Food Animal Residue Avoidance Databank (FARAD) for an extended meat and milk withdrawal interval.^23^

The limitations of this study include the small sample size and retrospective nature. In addition, 1 case did receive a dose of neostigmine, which could have affected hind‐gut motility and acted as a confounding factor to the effectiveness of lidocaine. The retrospective nature of this study limited the information that was available to analyze, and prevents a clear demonstration of efficacy because of the uncontrolled nature of the study. The small case number did not provide an accurate representation of the overall caseload from either of the 2 hospitals, and the conclusions drawn from the results are likely only specific to this set of animals. Overall, this case series demonstrated how lidocaine has been used as a prokinetic agent for the treatment of ileus in ruminants and camelids. The dosing regimen utilized in these cases did not appear to be associated with adverse events, and clinical outcomes of the reported cases were frequently positive.

## CONFLICT OF INTEREST DECLARATION

Authors declare no conflict of interest.

## OFF‐LABEL ANTIMICROBIAL DECLARATION

Authors declare no off‐label use of antimicrobials.

## INSTITUTIONAL ANIMAL CARE AND USE COMMITTEE (IACUC) OR OTHER APPROVAL DECLARATION

Authors declare no IACUC or other approval was needed.

## HUMAN ETHICS APPROVAL DECLARATION

Authors declare human ethics approval was not needed for this study.

## Supporting information


**Supplemental Table S1**: Descriptive summary of cases included in the retrospective analysis.
**Supplemental Table S3**: Ruminants and camelids whom underwent surgery with surgical findings.
**Supplemental Table S4**: Ancillary treatments for the ruminants and camelids included in the retrospective study. NSAIDs: nonsteroidal anti‐inflammatory.

**Supplemental Table S2**: Clinicopathologic and ultrasound findings of ruminants and camelids in the retrospective analysis. Normal reference ranges provided by Large Animal Internal Medicine.
