# Supplementary material for: Retrospective study on the use of lidocaine constant rate infusions for the treatment of ileus in ruminants and camelids
Source: J Vet Intern Med. 2021 Sep 13;35(6):2933–6. doi: 10.1111/jvim.16262 (PMC8692198; doi:10.1111/jvim.16262)
Supplement: Supplementary file 1 — Supplemental Table S1: Descriptive summary of cases included in the retrospective analysis. Supplemental Table S3: Ruminants and camelids whom underwent surgery with surgical findings. Supplemental Table S4: Ancillary treatments for the ruminants and camelids included in the retrospective study. NSAIDs: nonsteroidal anti‐inflammatory. [file JVIM-35-2933-s001.pdf]

Ruminant or Camelid	Presenting Complaint	Diagnosis	Duration of Hospital Stay (days)	Duration of Therapy (days)	Discharge Status
5 year old Saler Bull	Distended small intestines noted on rectal exam	Idiopathic cecal dilation and ileus	5	2	Discharged
1 year old mixed breed beef heifer	1 month duration of intermittent discomfort, ill-being and weight loss	Ileus	7	3	Discharged
5 year old Guernsey cow	Severe colic signs	Ceco-colic volvulus	3	1	Discharged
1 year old Angus Bull	Severe abdominal distention	Focal peritonitis	4	3	Discharged
1 day old Angus heifer	Hypothermia and unable to rise	Failure of passive transfer; ileus	5	1	Euthanized
1 year old female Suri Alpaca	Colic	Small intestinal intussusception	6	3	Discharged
8 year old mixed breed beef bull	Colic	Enteritis; spiral colon impaction	15	3	Discharged
3 day old Angus bull calf	Severe lethargy and suspected sepsis	Septicemia	3	1	Discharged
9 year old Myotonic doe	Bloat	Intestinal adhesions	7	3	Discharged
2.5 year old Angus bull	Colic; Abdominal distension	Ileus	4	1	Discharged

Supplemental Table 1: Descriptive summary of cases included in the retrospective analysis.

Ruminants and camelids	Surgery	Findings
5 year old Saler Bull	Abdominal Exploratory; Typhlotomies	Dilated cecum and large intestines due to abnormal band from the caudal greater omentum
1 year old mixed breed beef heifer	Abdominal Exploratory	Enlarged, fluid filled abomasum
5 year old Guernsey cow	Abdominal Exploratory	Ceco-colic volvulus
1 year old Angus Bull	Right Paramedian Abdominal Exploratory	Focal peritonitis with adhesions at the small intestines
1 year old female Suri Alpaca	Abdominal Exploratory; small intestinal resection and anastomosis	Intussusception
9 year old Myotonic doe	Abdominal Exploratory	Adhesions between small intestines and large intestines
2.5 year old Angus bull	Abdominal Exploratory	A firm band of tissues was found in the small intestines, but there was no evidence of torsion or volvulus

Supplemental Table 3: Ruminants and camelids whom underwent surgery with surgical findings.

**Ruminant or Camelid**	**Antibiotics**	**NSAIDs**	**Opioids**	**Others**
5 year old Saler Bull	Ceftiofur Hydrochloride; Ceftiofur Crystalline Free Acid	Flunixin meglumine	Butorphanol	Neostigmine CRI
1 year old mixed breed beef heifer	Ceftiofur Hydrochloride	Flunixin meglumine; Meloxicam		Ivermectin; Transfaunation
5 year old Guernsey cow	Ceftiofur Hydrochloride	Flunixin meglumine	Butorphanol	CMPK gel; Transfaunation
1 year old Angus Bull	Ceftiofur Sodium; Ceftiofur Hydrochloride; Ceftiofur Crystalline Free Acid; Tulathromycin		Butorphanol	Ketamine; Midazolam
1 day old Angus heifer	Florfenicol; Ampicillin	Flunixin meglumine		Clostridium Antitoxin
1 year old female Suri Alpaca	Ceftiofur Sodium; Ceftiofur Crystalline Free Acid; Tulathromycin	Flunixin meglumine; Meloxicam		Transfaunation
8 year old mixed breed beef bull	Ceftiofur Sodium; Florfenicol; Tulathromycin			Transfaunation; Plasma Transfusion; Furosemide; Diphenhydramine
3 day old Angus bull calf	Ceftiofur Sodium	Flunixin meglumine		Plasma Transfusion
9 year old Myotonic doe	Ceftiofur	Meloxicam		
2.5 year old Angus bull	Ceftiofur Hydrochloride	Flunixin meglumine; Meloxicam		

Supplemental Table 4: Ancillary treatments for the ruminants and camelids included in the retrospective study. NSAIDs: non-steroidal anti-inflammatory.
