# Supplementary material for: Retrospective study on the use of lidocaine constant rate infusions for the treatment of ileus in ruminants and camelids
Source: J Vet Intern Med. 2021 Sep 13;35(6):2933–6. doi: 10.1111/jvim.16262 (PMC8692198; doi:10.1111/jvim.16262)
Supplement: Supplementary file 2 — Supplemental Table S2: Clinicopathologic and ultrasound findings of ruminants and camelids in the retrospective analysis. Normal reference ranges provided by Large Animal Internal Medicine. [file JVIM-35-2933-s002.pdf]

Supplemental Table 2: Clinicopathologic and ultrasound findings of ruminants and camelids in the retrospective analysis. Normal Reference ranges provided by Large Animal Internal Medicine[1]

	Complete Blood Count					Biochemical Analysis					Venous Blood Gas					Ultrasound Findings at Presentation
**Patient**	**Segmented Neutrophil Count**	**Lymphocyte Count**	**Packed Cell Volume**	**Total Solids**	**Fibrinogen**	**Creatinine**	**Total Calcium**	**Chloride**	**AST**	**Total Bilirubin**	**Albumin**	**Bicarbonate**	**Ionized Calcium**	**TCO2**	**Lactate**	
5 year old Saler Bull	4.582	1.102	27	8.8	572	2	9.1	88	150	0.2	2.9	30				Distended cecum, distended loops of bowel
1 year old mixed breed beef heifer	4.611	3.219	42	7	254	1.1	9.1	86	57	0.3	3	34				Distended abomasum; hypomotile SI
5 year old Guernsey cow			35	8.8												Dilated cecum, distended loops of bowel
1 year old Angus Bull			50	7.4		3.4							0.8	>50		Enlarged gall bladder; motile intestines
1 day old Angus heifer			35	7.8								30.9	0.78	32	1.9	Non-motile SI; thickend SI wall; Free fluid within abdomen
1 year old female Suri Alpaca			26	6												Dilated small intestines; target lesion was observed
8 year old mixed breed beef bull	5.236	1.36	60	8.1	232	3	8.4	100	147	0.2	3.5	26		42		Hypomotile SI with thickened walls (13 mm); Omental edema
3 day old Angus bull calf	0.75	1.075	25	4.6								30.6	1.21	32.4	6.6	Hypomotile and fluid filled SI
9 year old Myotonic doe				6.2		1.4	7.8	97	1516	3.7	2.7	25				Distended abomasum; hypomotile SI
2.5 year old Angus bull			33	7		2		81						42	0.94	Distended loops of bowel; free fluid in abdomen
Normal Range (Cattle)	1.8-6.3 x 10^3	1.6-5.6 x 10^3	22-33%	6.7-7.46 g/dL	200-400 g/dL	1.0-2.0 mg/dL	9.7-12.4 mEq/L	97-111 mEq/L	78-132 IU/L	0.05-0.5 mg/dL	3-3.55 g/dL	17-29 mEq/L	<2 mmol/L	21-32 mEq/L	1.3-1.34 mmol/L	

1. Smith, BP. Large Animal Internal Medicine, Elsevier. St. Louis MO. 2015.
